# A large scale photonic matrix processor enabled by charge accumulation

**DOI:** 10.1515/nanoph-2022-0441

**Published:** 2022-10-28

**Authors:** Frank Brückerhoff-Plückelmann, Ivonne Bente, Daniel Wendland, Johannes Feldmann, C. David Wright, Harish Bhaskaran, Wolfram Pernice

**Affiliations:** Department of Physics, University of Münster, CeNTech, Heisenberg Str. 11, 48155 Muenster, Germany; Department of Physics, University of Münster, CeNTech, Heisenberg 11, 48149 Muenster, Germany; Department of Physics, University of Münster, CeNTech, Heisenberg 11, 48149 Muenster, Germany; Salience Labs Ltd, 46 Woodstock Rd, Oxford OX2 6HT, UK; University of Exeter, Faculty of Environment, Science and Economy, North Park Road, Exeter, UK; University of Oxford, Department of Materials, Parks Road, Oxford OX1 3PH, UK; University of Münster, Department of Physics, CeNTech, Heisenbergstraße 11, 48149 Münster, Germany

**Keywords:** matrix vector multiplication, photonic computing, time-multiplexing

## Abstract

Integrated neuromorphic photonic circuits aim to power complex artificial neural networks (ANNs) in an energy and time efficient way by exploiting the large bandwidth and the low loss of photonic structures. However, scaling photonic circuits to match the requirements of modern ANNs still remains challenging. In this perspective, we give an overview over the usual sizes of matrices processed in ANNs and compare them with the capability of existing photonic matrix processors. To address shortcomings of existing architectures, we propose a time multiplexed matrix processing scheme which virtually increases the size of a physical photonic crossbar array without requiring any additional electrical post-processing. We investigate the underlying process of time multiplexed incoherent optical accumulation and achieve accumulation accuracy of 98.9% with 1 ns pulses. Assuming state of the art active components and a reasonable crossbar array size, this processor architecture would enable matrix vector multiplications with 16,000 × 64 matrices all optically on an estimated area of 51.2 mm^2^, while performing more than 110 trillion multiply and accumulate operations per second.

## 1 Introduction

Matrix vector multiplications (MVMs) are the computational backbone of artificial neural networks (ANNs) as they mathematically describe the connections between neurons in the layers the network is composed of. Therefore, energy efficient, compact and high-speed matrix processors are crucial to power the complex ANNs deployed for autonomous driving [1] and language processing [2] among other important applications. Integrated photonic circuits are an attractive approach to implementing MVM processors due to their large optical bandwidth [3–6], inherent low latency [7] and minimal heating losses in comparison to electronic approaches. Prototypes deploying Mach–Zehnder interferometer (MZI) meshes [8], photonic crossbar arrays (PCAs) [9] and ring resonator weight banks [10] have demonstrated that photonic computing is viable and highlight the capabilities for MVM operations. However, one of the largest functional systems built to date is a 64 × 64 matrix processor deploying MZI meshes which is, even in view of being a substantial achievement, still of moderate size considering that modern ANNs consist of billions of free parameters. This particular system occupies a chip area of 150 mm^2^ [11]. Substantially increasing the size of such photonic circuits is challenging due to fabrication imperfections impacting the splitting ratios in the MZI meshes/crossbar arrays and the overall optical loss of the system. Therefore, a range of architectures and approaches are being developed which could enable processing larger ANNs in the photonic domain.

In this perspective, we give an overview on ANNs deployed in computer vision, natural language processing and combinatorial optimization and compare their requirements with the capability of different integrated photonic matrix processors. As a particular addition building on photonic processors, we propose a time multiplexed architecture that employs a high-speed reconfigurable photonic crossbar array to allow for MVM processing of larger matrices. Time multiplexing virtually increases the size of the PCA without suffering from the drawbacks of having to fabricate large photonic circuits. Moreover, this approach does not require any additional electrical processing. We experimentally characterize the process of time multiplexed incoherent optical accumulation and propose a design of the photonic circuit underlying the required matrix processor and estimate its performance.

## 2 Integrated photonics for artificial neural networks

On a basic level, artificial neural networks consist of linear matrix vector multiplications and non-linear activation functions. Additionally, their physical implementation requires memory and a process/data flow corresponding to the chosen ANN architecture. Integrated photonics promise to speed up ANNs in an energy efficient manner in two different ways. First, the photonic circuit computes the full ANN directly in one or few processing steps [12, 13]. Second, the photonic circuit only computes a part of the ANN, for example the MVMs [8], [9], [10], [11, 14, 15]. Only performing the MVMs drastically reduces the complexity of the photonic circuit and hence allows accelerating more complex ANNs, but this advantage comes at the cost of having to perform more electro-optic conversions.

**Figure 1:**
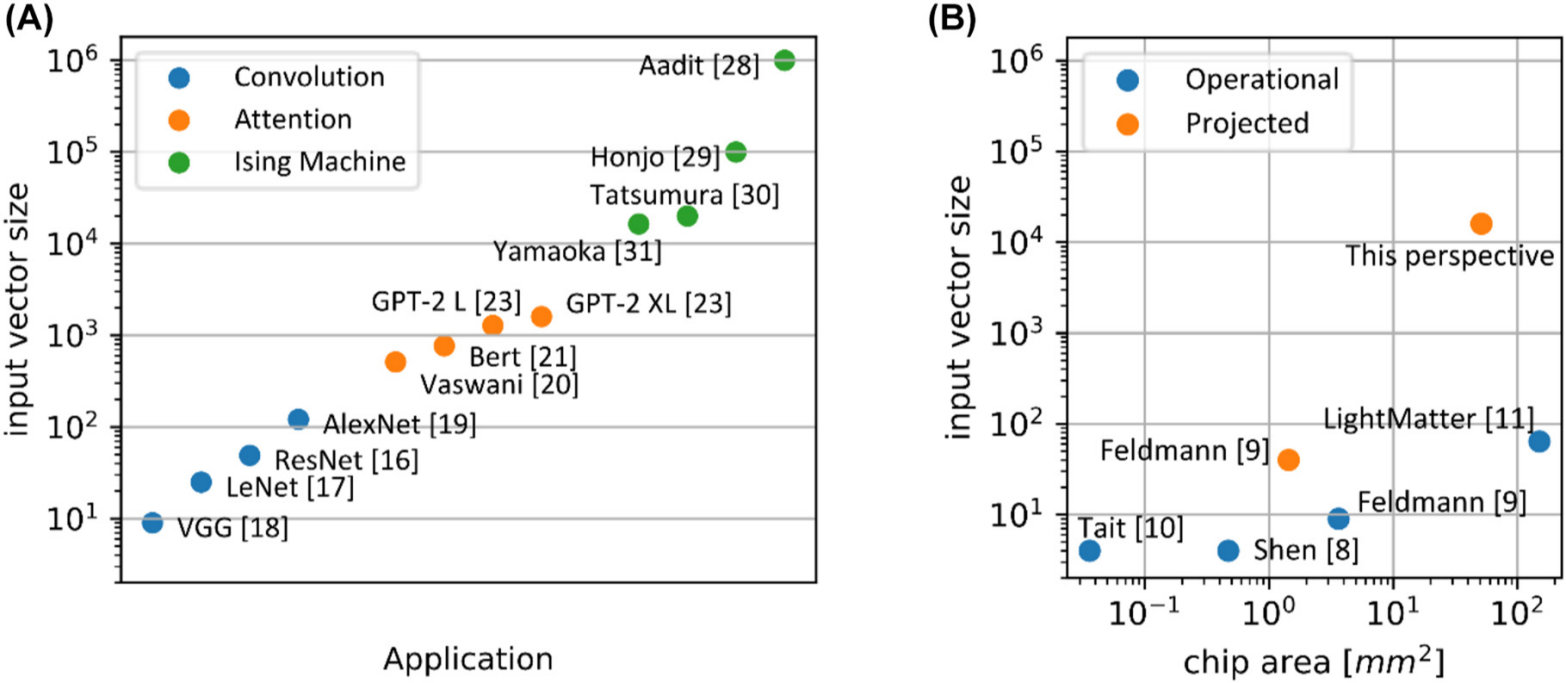
Applications and prototypes of (optical) neural networks. (A) Typical input vector size of the MVMs for different applications. Convolutional layers convolve the input with a kernel, typically in the range of 3 × 3 to 11 × 11 for computer vision. The attention head is the main block of the transformer architecture excelling in natural language processing. The input vector sizes depend on the model dimension, usually in the order of 10^3^. Hopfield and Boltzmann networks can mimic the Ising model and thus find good solutions to complex computational problems. The input vector size directly translates to the number of spins, which is in the range of 10^4^ to 10^6^ for modern Ising machines. (B) Input vector size of operational and projected integrated photonic matrix processors.

Figure 1A shows the typical input vector sizes of the MVM processors depending on the application. Convolutional neural networks perform excellently in computer vision. The main building blocks are convolutional layers which calculate the convolution of the layer input with a trained kernel. The employed kernels are typically rather small, ranging from 3 × 3 to 11 × 11 for many networks [16–19], which in turn leads to small input vector sizes for one channel. In contrast, transformer architectures excel in the area of natural language processing. Their main feature is the attention module, which computes correlations between the different symbols in the input sequence. The input vector size of the performed MVMs depends on the model dimension which is often on the order of 10^3^ [20–23]. Finally, recurrent neural networks like Hopfield nets and Boltzmann machines can find the ground state of the Ising model and hence are suitable to obtaining good solutions of NP-hard problems like the travelling salesman problem [24–27]. The input vector size of the underlying MVMs directly translates to the number of spins in the ising model, where state of the art ising machines can simulate spins in the order of 10^4^ to 10^6^ [28–31].

In contrast, Figure 1B shows the (projected) input vector size of various integrated photonic matrix processors. In photonic computing, the input vector is encoded in optical pulses and the respective multiplications are carried out via interaction of the pulses with phase-shifters, attenuators, or amplifiers [8, 32, 33]. There are two different approaches to accumulate the weighted optical pulses, performing either a coherent, phase sensitive superposition [8] or performing an incoherent superposition [9]. While coherent superposition also enables subtraction via destructive interference, practical systems require phase error compensation. In contrast, when using incoherent signals, two pulses of different wavelength are temporally overlapped to perform an incoherent superposition. Since the result of the MVM is calculated at the photodetectors, all interference effects are averaged out if the frequency detuning between both pulses is larger than the detector bandwidth [34]. The main drawback of incoherent superposition is that subtraction cannot be performed optically. The advantage is that the overall system is much more tolerant to fabrication imperfections and measurement conditions because of the inherent phase insensitivity. Furthermore, incoherent superposition can be easily used in combination with wavelength division multiplexing (WDM) [35]. However, both approaches are using a physical photonic circuit to represent the matrix vector multiplication leading to huge circuits for large scale MVMs. A common workaround is to deploy tiled matrix multiplication to reduce the matrix size; however this creates additional electronical overhead. In this perspective we propose an architecture that virtually increases the size of the photonic matrix processor based on time multiplexed incoherent optical accumulation. This allows computing MVMs with large input vector size all optically (in contrast to electronic addition of tiles, which require much more analogue to digital conversions and additional electronic circuitry) on a chip size compatible with commercial foundry processes.

## 3 Time multiplexed incoherent optical accumulation

**Figure 2:**
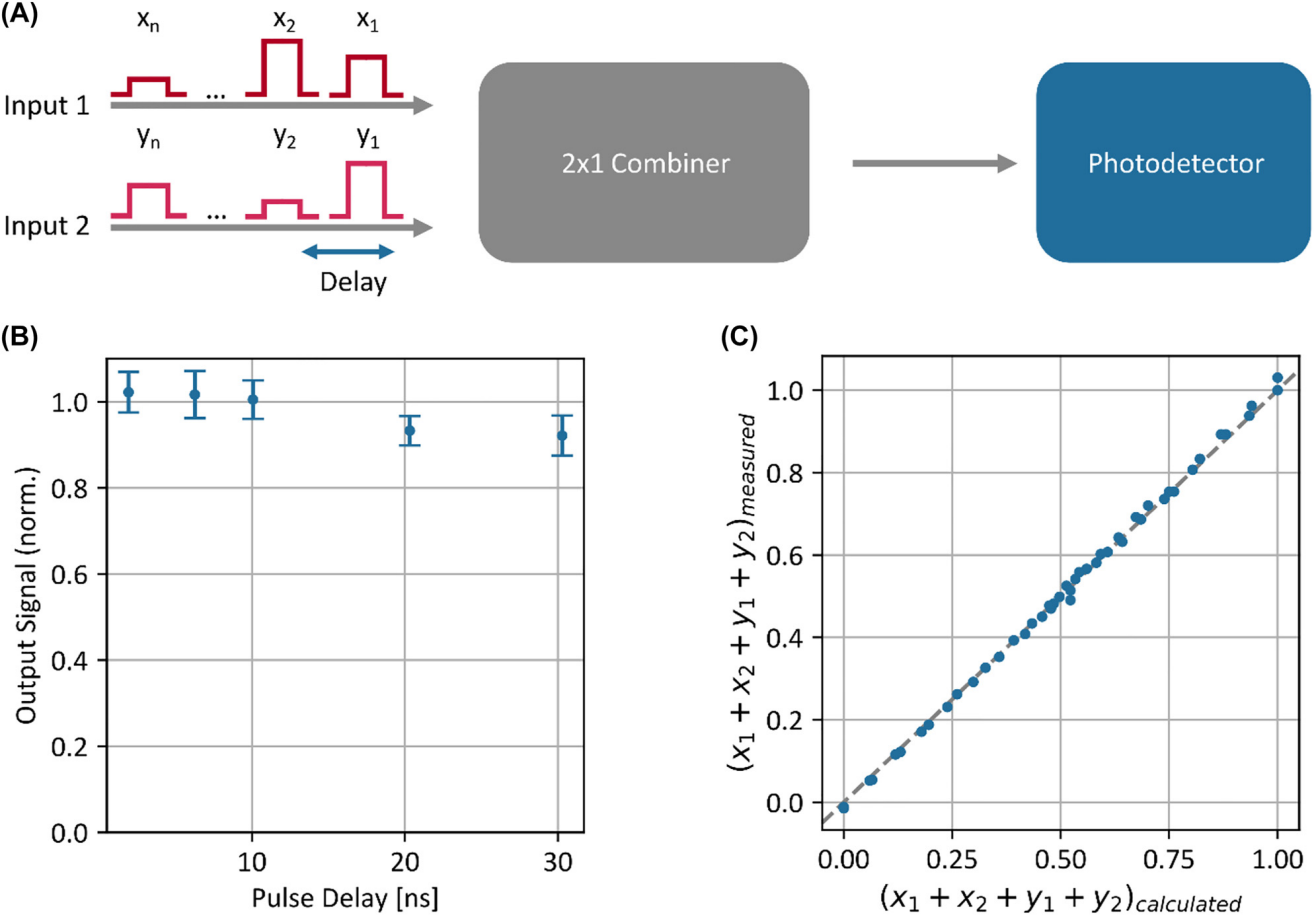
Time multiplexed incoherent accumulation. (A) Concept of time multiplexed incoherent accumulation. We combine two accumulation schemes, temporally overlapping pulses of different wavelength and charge accumulation inside a detector by delayed pulses. Both processes are phase insensitive. (B) Accumulation time of a 10 MHz Photodetector. We determine the accumulation time of the photodetector by comparing the detector signal of the delayed accumulated pulse with the signal of the individual pulses. Up to a delay of about 10 ns the error induced by this accumulation scheme is within the uncertainty of the measurement setup. (C) Accuracy of time multiplexed incoherent accumulation. We accumulate four different 1 ns pulses as sketched in (A) with a delay of 1.86 ns. The measured signal is distributed around the calculated signal with a standard deviation of 1.1% which is comparable the noise within the measurement setup itself.

In order to virtually enlarge the processing capacity of photonic MVM processors, we combine the concept of incoherent superposition with charge accumulation inside a photodetector (Figure 2A). We perform incoherent superposition by temporally overlapping two pulses of different wavelengths and accumulate several pulses of the same wavelength by making use of charge accumulation inside the photodetector [36]. If the delay between the pulses is short in comparison to the inverse detector bandwidth, the detector cannot distinguish between the individual pulses. Instead, the output signal of the detector has higher amplitude. To show the feasibility of this approach experimentally, we use 1 ns pulses at 1550 and 1560 nm together with a 10 MHz detector (New Focus Model 2053) to characterize the process of time multiplexed incoherent optical accumulation. Figure 2B shows the detector output signal of two delayed pulses normalized to the manually summed output signal of the individual pulses for different delays between the pulses. We perform each individual measurement 10 times, where the measurement setup itself shows a noise level in the order of 1–2%. Up to a delay of 10 ns, the error caused by charge accumulation inside the detector is within the uncertainty of the measurement setup itself. Next, we perform time accumulation simultaneously with incoherent superposition. We accumulate four pulses of two different wavelengths with a time delay of 1.86 ns in each wavelength channel. Figure 2C shows the measured sum of the four pulses versus the calculated sum of the pulses. The measured signals are distributed around the ideal line with a standard deviation of 1.1% which is again inside the uncertainty of the measurement itself. The main advantage of this approach is the scaling properties. Individual approaches, incoherent superposition and charge accumulation, scale linearly with the number of wavelength channels/pulses. However, the combination of both scales with the number of wavelength channels × number of pulses. Moreover, the comparably slow detector and readout process enables low noise operation which in combination with high saturation powers makes scaling possible (in our experiments, the deployed detector has a noise equivalent power of 0.34 pW/Hz and the saturation power is 10 mW). For broadband operation, the wavelength dependent responsivity of the detector can be compensated by setting a wavelength dependent calibration factor.

## 4 Large-scale photonic matrix vector multiplication

**Figure 3:**
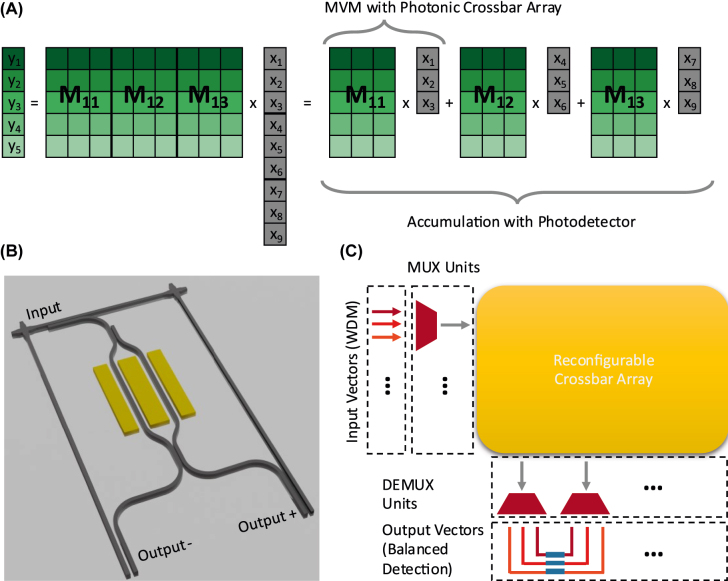
Large scale photonic matrix vector multiplication scheme. (A) Time multiplexed matrix vector multiplication (MVM). We decompose the large-scale matrix vector multiplication into several small MVMs which the photonic crossbar array computes. We sum the intermediate results by charge accumulation inside the photodetector. In this way, no additional electrical processing is required. (B) Concept of an active, arbitrary valued matrix cell. We design matrix cells deploying an MZM as the matrix weight. In this way, the weight can be modulated as fast as the input vector, which is crucial for this matrix architecture. Moreover, the MZM allows for lossless reference computation. (C) Sketch of the complete matrix processor. The processor computes several MVMs in parallel by encoding each vector in a different wavelength range. Before sending the vectors to the crossbar array, they are multiplexed together and afterwards demultiplexed again to obtain the individual output vectors. Finally, balanced detection is performed to obtain the correct result.

Since the accumulation is independent from the multiplication for incoherent crossbar arrays, we can directly transfer the concept of time multiplexed optical accumulation to the complete system. In this way, we virtually increase the size of a photonic crossbar array beyond its physical dimensions without inducing any additional electrical processing. Figure 3A depicts how the matrix multiplication *y* = *M* ⋅ *x* is performed. In the context of artificial neural networks, each component of *x* corresponds to the activation of a neuron, thus we assume *x*_*i*_ ≥ 0. MVMs with arbitrary input vectors can be computed by using a reference vector component [35]. In contrast, the weights connecting the various neurons of the ANN can be positive and negative. Hence, *M* is an arbitrary real valued *m* × *n* matrix. The *m* × *n* matrix is divided into several *m* × *n*′ matrices with *n*′ ≪ *n*. Then, the matrix vector multiplication is performed stepwise where the first sub matrix is multiplied with the first elements of the vector, analogously for the other sub matrices. The different sub results are then accumulated by the photodetector to obtain the correct result without requiring any electro-optical conversion and electrical processing. In this way, only a *m* × *n*′ crossbar array is required to carry out the same processing as obtained from the significantly larger *m* × *n* matrix, greatly reducing the complexity of the photonic circuit.

We propose a design for a matrix cell of the photonic crossbar array which encodes the matrix weight into a Mach–Zehnder modulator (MZM) as shown in Figure 3B. This enables a fast modulation of the matrix elements which is crucial for the time multiplexed MVMs. Moreover, it allows for reference computation without inducing any additional loss by performing balanced detection between the two output waveguides. In this scheme, a matrix weight of zero is encoded by setting the MZM to equal splitting between Output+ and Output−. Similarly, negative weights are implemented by guiding are larger fraction of the Input to Output- and vice versa. State of the art MZM achieves modulation speeds of 100 GHz on a comparably small footprint [37–40]. Assuming the silicon-organic hybrid MZM in [40], the crossbar cell size would be in the order of 0.05 mm^2^.

Figure 3C sketches the photonic circuit of the complete system consisting of MZMs to modulate the input vectors, a quickly reconfigurable photonic crossbar array, comparably slow photodetectors for temporal accumulation and wavelength multiplexer. Wavelength division multiplexing enables several parallel computational channels which further increases the speed of the photonic matrix processor [35]. We estimate the ultimate performance capabilities of the time multiplexed matrix processor architecture based on the characterized 10 MHz photodetector, 100 GHz MZMs and a 16 × 64 crossbar array. The photodetector precisely accumulates the optical pulses within a time of 10 ns, corresponding to 1000 pulses due to the modulation speed of the MZMs. In this way the size of the crossbar array is virtually increased to 16,000 × 64 but only requires 1024 MZMs instead of more than a million as its equivalent physical counterpart. Moreover, it greatly reduces the overall loss of the system in comparison to a physical, incoherent PCA of the same size, since the splitter induced loss scales as 1/*n* where n is the number of physical input waveguides to the PCA. Even though the time-multiplexing decreases the theoretical maximal speed of the system (due to the need for slower than state-of-the-art photodetectors), wavelength division multiplexing still unlocks exceptional computational power. Frequency combs can generate the required input wavelengths over a wavelength range of 1500–1650 nm [9]. Assuming a 100 GHz spacing between the wavelengths of one channel to avoid interference, each optical matrix vector multiplication requires 16 (#inputs) x 0.8 nm (#channel spacing) = 12.8 nm optical bandwidth, so 11 vectors can be computed in parallel. The matrix processor performs MVMs with 16,000 × 64 matrices at a speed of 10 MHz on an estimated crossbar array area of 51.2 mm^2^, leading to 112.64 trillion multiply and accumulate operations per second. The main advantage of this approach is that the matrix vector multiplication is performed fully optically without any intermediate electrical processing steps. A physical implementation would thus greatly reduce the bottleneck of optical data processing caused by electro optical conversion.

## 5 Summary

Photonic computing is a promising approach to fulfil the ever-growing demand on computational performance arising from the use of artificial neural networks. The combination of wavelength division multiplexing and in-memory computing enables matrix-vectors-multiplications at unprecedented computation speeds and low latency times [7, 9, 41]. However, scaling the photonic circuit to perform large-scale MVMs remains challenging, due to the physical size of the photonic components and fabrication imperfections. We propose a novel computation scheme for photonic matrix processors, which allows one to virtually increase the size of the MVMs without suffering from the drawbacks of large photonic circuits. The computation scheme uses time multiplexed incoherent accumulation, which encodes the pulses in both the frequency and time domain. We characterize this scheme with a 10 MHz detector and 1 ns pulses, allowing integration in the time domain up to 10 ns with an accuracy of 98.9%. Assuming a 100 GHz MZM [40], a 1500–1650 nm frequency comb [9] and a reasonable sized physical 16 × 64 crossbar [11], the system could perform MVMs with 16,000 × 64 matrices all optically at a speed above 110 trillion multiply and accumulate operations per second.
